# Is atypical parathyroid tumor a different clinical entity than parathyroid adenoma and carcinoma? A retrospective review of a large single-center case series

**DOI:** 10.1007/s13304-025-02445-1

**Published:** 2025-10-29

**Authors:** C. Maconi, A. M. Saibene, L. Castellani, P. Lozza, C. Pescia, M. Falleni, L. De Pasquale

**Affiliations:** 1 Università degli Studi di Milano, Milan, Italy; 2 Asst Santi Paolo e Carlo, Ospedale San Paolo Milano, Milan, Italy

**Keywords:** Primary hyperparathyroidism, Atypical parathyroid tumor, Parathyroid adenoma, Parathyroid carcinoma, Parathyroidectomy

## Abstract

Primary hyperparathyroidism is primarily caused by single-gland pathology (80–85% of cases). According to the 2022 World Health Organization (WHO) guidelines (Erickson et al. in Endocr Pathol, 2022), single-gland pathologies include parathyroid adenoma, atypical parathyroid tumor and parathyroid carcinoma (Gurrado in J Clin Med 12:6297, 2023). The aim of this study is to identify differences or similarities of both pre-, intra- and post-operative characteristics between atypical parathyroid tumor and parathyroid adenoma/carcinoma, thereby establishing an appropriate follow-up protocol for atypical parathyroid tumor. We retrospectively analyzed 437 patients who underwent parathyroidectomy for primary hyperparathyroidism between 2012 and 2022 at the Thyroid Unit of ASST Santi Paolo e Carlo in Milan focusing our analysis on 352 patients with single-gland disease. Several pre-, intra-, and post-operative variables, including follow-up, were analyzed and compared using non-parametric statistical methods. Histological analysis identified 316 cases of PA (90%), 27 cases of atypical parathyroid tumor (7.7%), and 9 cases of parathyroid carcinoma (2.3%). Patients with atypical parathyroid tumor had significantly higher pre-operative PTH levels, intermediate calcium levels, falling between those of parathyroid adenoma and parathyroid carcinoma patients and larger gland diameter. No cases of disease persistence or recurrence were observed in patients with atypical parathyroid tumor after a mean follow-up of 42.8 months. APT exhibits biochemical and pathological features overlap with both PA and PC. However, the lack of recurrence or persistence suggests that APT behaves more similarly to PA than PC. The favorable evolution of APT in our case series could be a factor in favor of reducing the follow-up time for atypical tumors to a shorter period than the one recommended for carcinomas.

## Introduction

Primary hyperparathyroidism (PHPT) is a common endocrine disorder, consisting of parathyroid hormone overproduction by one or more parathyroid glands (PGs) leading to high or inappropriate serum calcium level. Clinically, PHPT can be classified into two major forms: symptomatic and asymptomatic. In Italy, the majority of cases are currently identified as asymptomatic (approximately 80%), most often detected incidentally through routine biochemical testing in patients without evident clinical manifestations. Asymptomatic PHPT may be further classified as complicated and uncomplicated forms, depending on the silent target-organ involvement.

Symptomatic PHPT (around 20% of cases) is characterized by clinical manifestations related to PTH overproduction and hypecalcaemia. The most important renal complications are nephrolithiasis (15–20%) and hypercalciuria (defined as 24-h urinary calcium excretion > 250 mg in women and > 300 mg in men), which is one of the main risk factors for stone formation. PHPT has also been implicated in the progressive decline of renal function, potentially contributing to chronic kidney disease. Bone complications are equally significant: patients frequently present with osteoporosis and reduced Bone Mineral Density (BMD), as assessed by dual-energy X-ray absorpiometry (MXA). The reduction predominantly affects cortical bone, whereas trabecular bone is generally less compromised [1].

Sporadic primary hyperparathyroidism (PHPT) is primarily caused by single-gland pathology in approximately 80–85% of cases, while in 15–20% of cases, multiple parathyroid glands (PGs) are involved.

According to the 2022 World Health Organization (WHO) guidelines [2], single-gland pathologies include parathyroid adenoma (PA), atypical parathyroid tumor (APT) and parathyroid carcinoma (PC).

APT is defined as a neoplasm with uncertain malignant potential. In the recent update of the WHO 5th edition of endocrine tumor, this current definition replaces the previous term of *atypical parathyroid adenoma,* to emphasize its uncertain malignancy [3].

According to published data, the incidence of atypical parathyroid tumor ranges from 0.5 to 4.4% with a female preponderance [4].

Microscopically, it’s mainly composed of chief cells and displays some histological features typical of PC, such as adherence to adjacent structures, monotonous sheet-like or trabecular growth, cytologic atypia, fibrosis (including band-like fibrosis), cells extending into but not through the capsule and high mitotic activity. Despite these worrisome features, however, APT lacks unequivocal capsular, vascular, or perineural invasion, or invasion into adjacent structures, or documented metastatic disease [2]. In summary, the diagnosis of APT is applied to parathyroid neoplasms that exhibit significant atypical features suggestive of parathyroid carcinoma but lack definitive evidence of invasive growth.

Additionally, Abbona et al. [5] conducted a retrospective analysis to evaluate differences in the proliferative index of parathyroid lesions responsible for primary hyperparathyroidism using the Mib1-Ki67 antibody. Their findings indicated that a Ki67 index greater than 6% was associated with aggressive tumor behavior, including both local invasion and distant spread. Notably, a high percentage of Ki67-positive cells was a characteristic feature of parathyroid carcinomas, which exhibit a higher proliferation rate, whereas adenomas did not demonstrate this pattern. Conversely, lesions with a Ki67 index below 5% were indicative of benign behavior, a finding supported by other authors. A specific immunohistochemical marker for APT has not yet been identified, so, the diagnosis of ATP is based exclusively on histopathological criteria [6].

Unfortunately, a specific immunohistochemical marker for APT has not yet been identified, and, therefore, the diagnosis of ATP is based exclusively on histopathological criteria in the appropriate clinical context.

The aim of this study is to identify differences or similarities of both pre-, intra- and post-operative characteristics, between atypical parathyroid tumor and parathyroid adenoma/carcinoma, thereby establishing an appropriate follow-up criteria for atypical parathyroid tumor.

## Methods

We conducted a retrospective review of clinical data from 437 patients who underwent parathyroidectomy for PHPT between 2012 and 2022 at the Thyroid Unit of ASST Santi Paolo e Carlo in Milan. Of these, we included only 352 patients who underwent surgery for PHPT due to single-gland disease, while 85 patients with multiglandular involvement were excluded.

Surgery was performed by two experienced endocrine surgeons, and the samples were always analyzed by the same pathologist specialized in parathyroid glands.

The following informations were collected for each patient affected by primary hyperparathyroidism due to single-gland disease and undergoing parathyroidectomy (PTX): sex, age at the time of surgery, clinical manifestations, pre-operative values of PTH, phosphorus, calcium, pre-operative imaging studies, modality of intervention, calcium levels in the first two post-operative days, post-operative PTH, weight and diameter of the excised glands, post-operative complications such as transient and permanent hypoparathyroidism, hemorrhage, transient or permanent recurrent nerve injury, Hungry Bone Syndrome, and follow-up in terms of recurrence and persistence. In our retrospective study, we adopted the definition of persistent and recurrent PHPT according to ISS/AME/SIOMMMS Guidelines (2023). Persistent PHPT is defined as the persistence of biochemical evidence of PHPT, with hypercalcemia detectable within the first 6 months after PTX. Recurrent PHPT is defined as the reappearance of a biochemical profile with hypercalcemia after initially successful PTX intended as the maintenance of normal serum level of calcium and PTH levels for at least 6 months post-operatively.

Calcium levels were expressed in mg/dl (reference range 8.4–10.2 mg/dl), PTH in pg/ml (reference range 8.7–79.6 pg/ml) and phosphorus in mg/dl (reference range 2.5–4.5 mg/dl).

Post-operative hypocalcemia was defined as serum calcium levels < 8.4 mg/dl with PTH values within the normal range, while hypoparathyroidism was defined as a condition in which calcium and PTH are below the lower limit. According to the reference ranges of our laboratory, patients with PTH levels below 8.7 pg/ml and serum calcium < 8.4 mg/dL were classified as having hypoparathyroidism. Both complications were considered transient if they resolved within the first 6 months after surgery and permanent if the abnormal values persisted for more than 6 months post-surgery.

These variables, including follow-up, were analyzed and compared using non-parametric statistical methods.

For statistical analysis, two types of tests were employed. The Chi-Square test was used to compare the frequency in the different histology groups of binomial variables, such as gender distribution, concordance with pre-operative imaging, and frequency of post-operative complications, hypoparathyroidism, hemorrhage, recurrent nerve injuries, and Hungry Bone Syndrome.

For continuous variables, given the uneven distribution of cases in the different histology groups and the small number of PC, we did not assume normal distribution and employed non-parametric tests.

More specifically, the Kruskal–Wallis test was used to compare the different histology groups in terms of all other study parameters (patient age at surgery, pre-operative calcium, phosphorus, PTH, and creatinine levels, diameter and weight of excised glands, calcium levels on the first and second post-operative days, and post-operative PTH).

Statistical analyses were performed using SPSS software (PASW Statistics for Windows, version 21.0; SPSS Inc., Chicago, IL).

The significance level was set at *p* < 0.05, and all tests were two-tailed.

Before surgery, each patient underwent neck ultrasound and scintigraphy with radiopharmaceutical (Tc 99 MIBI). In case of traditional imaging (ultrasounds and SestaMIBI scintigraphy) discordance or negativity our patients underwent PET-TC (^18^F-choline).

In cases of suspected single-gland disease, as suggested by pre-operative localization studies and in the absence of concomitant thyroid pathology, a minimally invasive video-assisted parathyroidectomy (MIVAP) with intraoperative PTH (I.O. PTH) assay was performed. When localization was unreliable and/or concomitant thyroid disease was present, a conventional neck exploration with I.O. PTH assay was undertaken. In patients with suspected parathyroid carcinoma (PC), an en-bloc resection of the parathyroid lesion together with the ipsilateral thyroid lobe was carried out, always with I.O. PTH assay. In patients treated with traditional cervical exploration, in case of significant I.O. PTH decrease and in the absence of thyroid pathology of the opposite lobe, the approach was unilateral, without exploration of the other side.

All patients also underwent post-operative laryngoscopy to evaluate vocal cord motility after surgery; cases of recurrent nerve paralysis were classified as transient or permanent depending on whether vocal cord paralysis and vocal alteration persisted for more or less than 12 months post-surgery.

For PC and APT, follow-up assessments were performed at 3, 6, and 12 months during the first postoperative year, and at 6 months during the second year. Subsequently, patients were visited once a year for at least 10 years. Each visit included serum measurements of calcium, phosforyus, PTH, and 25-OH vitamin D, as well as a neck ultrasound examination. As no recurrences were detected, no additional imaging investigations were required. The mean follow-up time for patients with APT was 42.8 months and for patients with PC was 74.4 months.

## Results

**Fig. 1:**
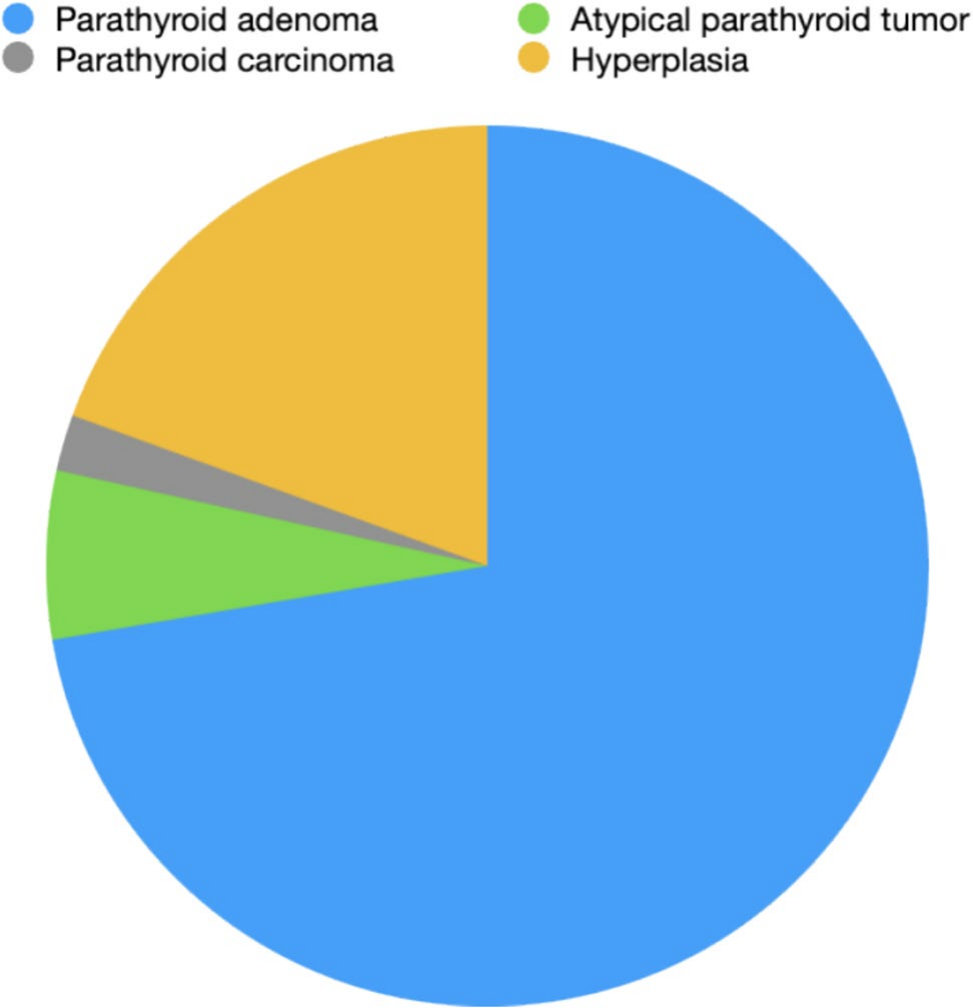
Distribution of histological diagnoses among patients with primary hyperparathyroidism

The final histological analysis of 437 patients revealed 316 cases of PA (72.3%), 27 of APT (6.2%), 9 of PC (2.1%) and 85 of hyperplasia (19.4%) (Fig. 1).

### Demographic data

Patients with APT (*n* = 27) had a mean age of 64.5 ± 26 (20–84) years, compared with 73.5 ± 32.5 years (39–76) in PC and 62 ± 16.25 years (32–79) in PA. Althought the mean age was higher in patients with PC (73.5 ± 32.5 years), the difference was not statistically significant [Kruskal–Wallis test *p* = 0.77]. The gender distribution among the analyzed patients showed a statistically significantly higher incidence of APT and PA in females, whereas PC was more frequent in men [Chi-Square test *p* = 0.033] (Table 1).

**Table 1:** Demographic characteristics of patients according to histological diagnosis of parathyroid adenoma, atypical parathyroid tumor and parathyroid carcinoma

	APT *N* = 27	PC *N* = 9	PA *N* = 316
Age	64.5 ± 26 years (20–84)	73.5 ± 32.5 years (39–76)	62 ± 16.25 years (32–79)
Female	18 (66.67%)	4 (44.44%)	246 (77.85%)
Male	9 (33.33%)	5 (55.56%)	70 (22.15%)

### Symptoms and comorbidities

About 27 APT, 8 patients were found to be asymptomatic (29.63%), 12 patients had underlying kidney involvement (44.44%), 4 had bone involvement (14.82%), 3 had neurological symptoms (11.11%). No patients with APT had hypertension or gastrointestinal involvement.

Among the eight asymptomatic patients, in seven cases hypercalcemia was detected during blood tests performed for other reasons, while one showed focal neck uptake on an FDG-PET scan performed for another indication. A targeted neck ultrasound in this patient demonstrated a possible pathological parathyroid corresponding to the area of uptake, and subsequent biochemical evaluation confirmed severe PHPT with markedly elevated serum calcium levels. All 12 patients with renal involvement presented with symptomatic urinary lithiasis. Among the four patients with skeletal involvement, three exhibited severe osteoporosis and one had brown tumors. Finally, the three patients with neurological manifestations presented cognitive decline.

Among the nine patients with PC, 2 (22.22%) were asymptomatic and hypercalcemia was detected during blood tests performed for other reasons; 3 (33.33%) presented with symptomatic urinary lithiasis, and 1 with a brown tumor of the tibia and femur. Of the 3 (33.33%) patients with neurological manifestations, 1 (11.12%) presented to the emergency department in a soporous state, while two showed cognitive decline and depression.

**Fig. 2:**
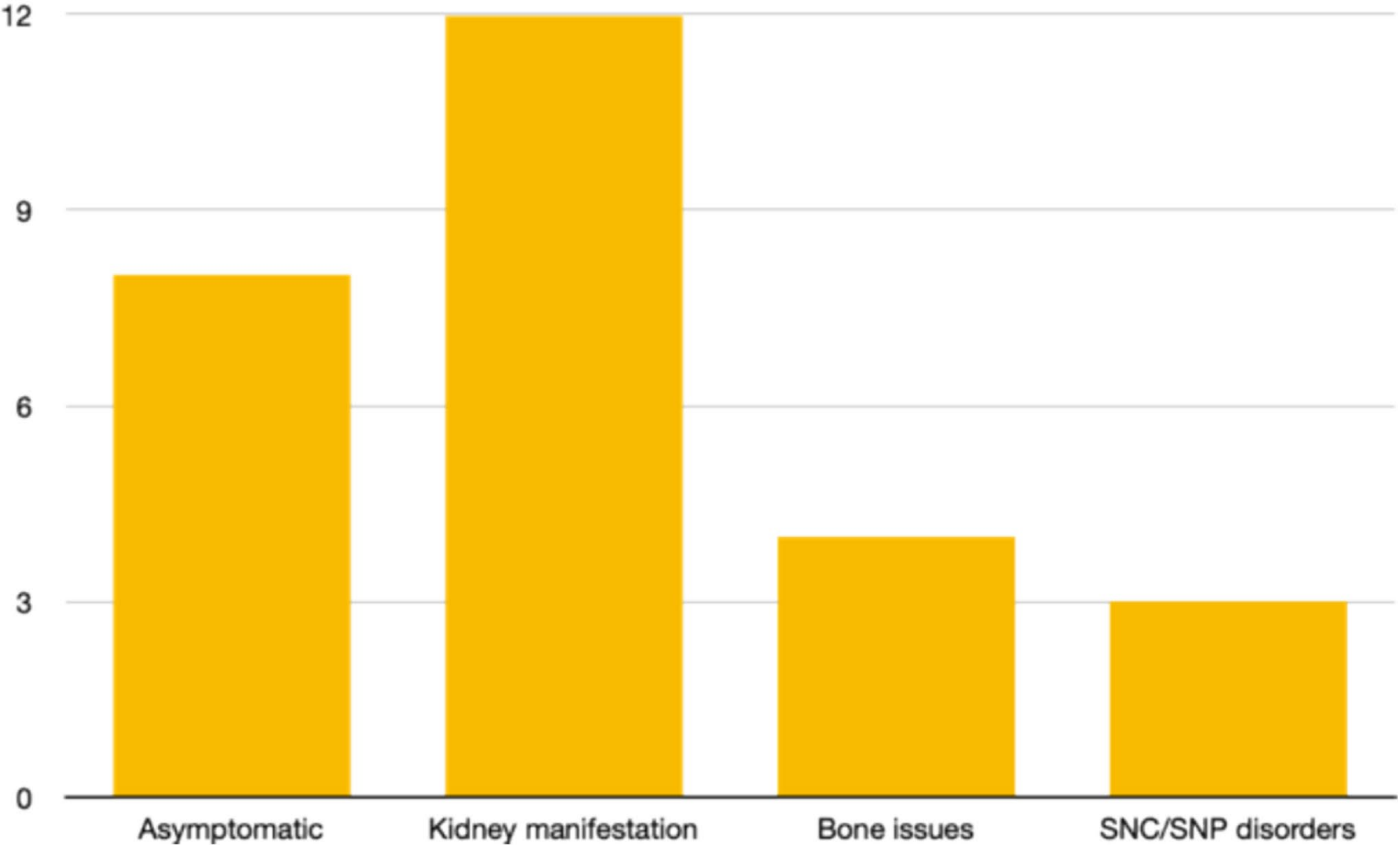
Distribution of clinical manifestations among patients with atypical parathyroid tumor

Among the 316 patients with PA, all those with renal involvement had symptomatic urinary lithiasis, and all asymptomatic patients were diagnosed incidentally through elevated calcium levels on blood tests performed for another indications. Patients with skeletal involvement uniformly presented with osteoporosis. Of the 27 patients with neurological symptoms, 21 had depression and 6 cognitive decline. The most frequent gastrointestinal manifestation was vomiting (Fig. 2).

APT group showed higher rate of asymptomatic patients (29.63% vs 22.22% vs 27.22%) and kidney involvement (44.44% vs 33.33% vs 31.65%) than PC and PA, although these differences were not statistically significant [Chi-square test, respectively *p* = 0.23 and *p* = 0.34] (Table 2).

**Table 2:** Distribution of symptoms and organ involvement in the three histological groups

	APT *N* = 27	PC *N* = 9	PA *N* = 316
Kidney involvement	12 (44.44%)	3 (33.33%)	97 (30.69%)
Asymptomatic	8 (29.63%)	2 (22.22%)	83 (26.27%)
Bone involvement	4 (14.81%)	1 (11.11%)	77 (24.37%)
Neurological symptoms	3 (11.11%)	3 (33.33%)	27 (8.54%)
Gastrointestinal involvement	0	0	23 (7.28%)
Cardiovascular symptoms (hypertension)	0	0	9 (2.85%)

About comorbidities, we showed that 4 APT patients had concomitant thyroid disease (18.81%), 48 PA patients (15.19%) and 2 PC patients (22.22%), again with a non-statistically differences [Chi-Square test *p* = 0.844].

### Pre-operative data

Regarding preoperative imaging, concordance between the tests performed was observed in 21 of 27 patients in the APT group (77.8%), in PC group in 6 patients (66.67%) and in the PA in 184 patients (58.23%), with no significant differences between groups [Chi-Square test *p* = 0.127].

**Fig. 3:**
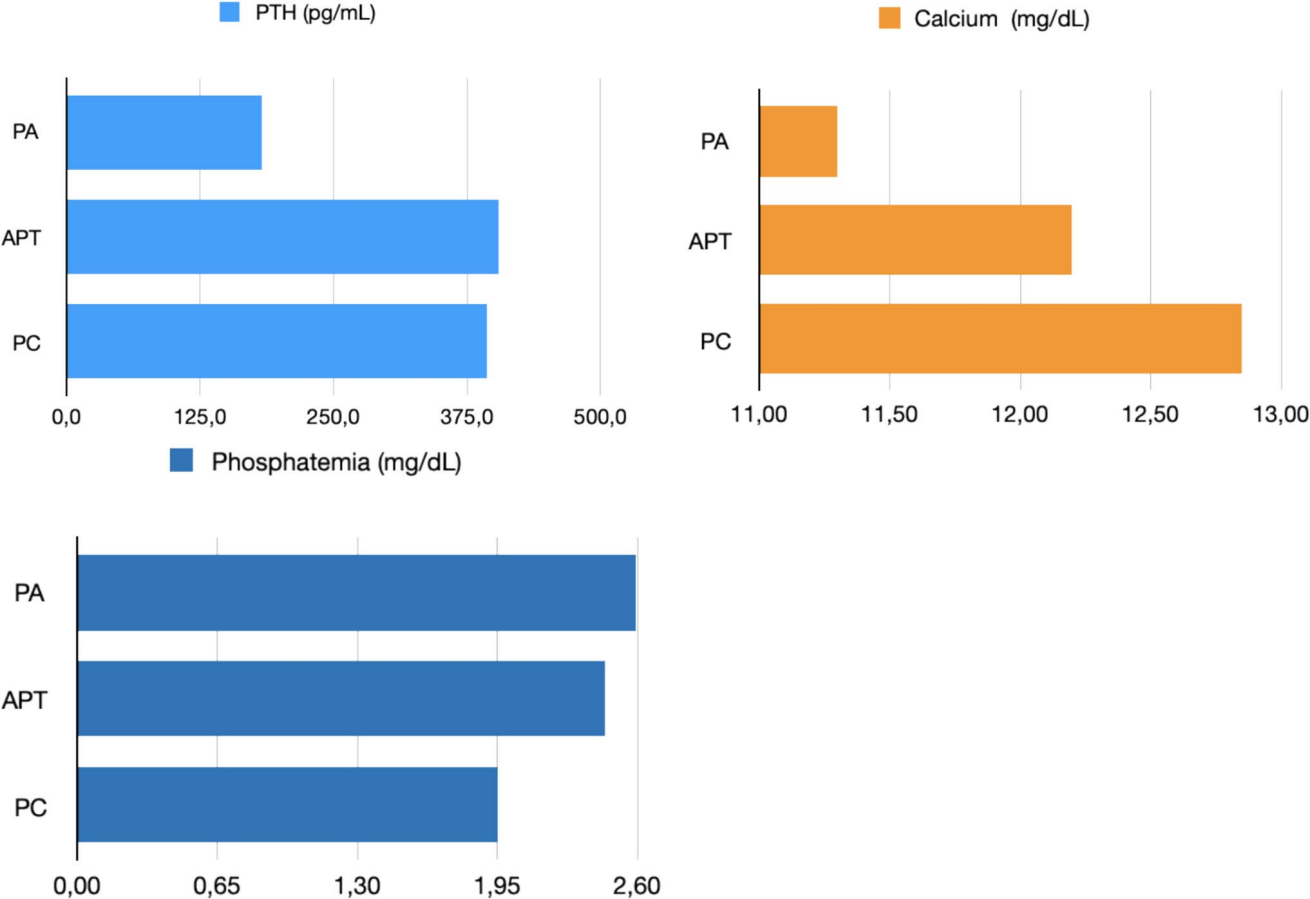
Pre-operative serum calcium, phosphorus, and PTH levels in patients with atypical parathyroid tumor

For APT, the mean preoperative calcium level was 12.2 ± 0.975 (10.3–14.8) mg/dl, the mean preoperative phosphorus level was 2.45 ± 0.675 (1.6–1.9) mg/dl and the mean PTH level was 404.5 ± 283.95 (112–957) pg/ml. Complete calcium, phosphorous and PTH levels for all groups are reported in Table 3 and in Fig. 3. Pre-operative PTH levels were significantly higher in patients with APT compared to those with PA and PC [Kruskal–Wallis test *p* < 0.001]. For calcium level, patient with PC exhibited the highest values, while those with APT had intermediate calcium levels, falling between those of PA and PC patients, again with a statistically significant difference [Kruskal–Wallis test *p* < 0.001]. Last, patients with PC had the lowest phosphate levels, although the statistical significance was minimal [Kruskal–Wallis test *p* = 0.049].

**Table 3:** Pre-operative biochemical parameters in patients with atypical parathyroid tumor, carcinoma, and adenoma

	APT *N* = 27	PC *N* = 9	PA *N* = 316
Preoperative calcium level (mg/dl)	12.2 ± 0.975 (10.3–14.8)	12.85 ± 2.5375 (12.5–18)	11.3 ± 0.8 (8.9–16.5)
Preoperative phosphorus level (mg/dl)	2.45 ± 0.675 (1.6–1.9)	1.95 ± 0.425 (1.7–2.5)	2.6 ± 0.4225 (1.3–3.8)
Preoperative PTH level (pg/ml)	404.5 ± 283.95 (112–957)	393.5 ± 1271.25 (250–2160)	183 ± 156.9 (73.1–1861)

### Operative data

Among 27 patients with APT, 17 (62.96%) underwent MIVAP, 3 (11.11%) traditional parathyroidectomy (two for large gland size and one due to negative preoperative imaging), and 4 (14.81%) traditional parathyroidectomy combined with thyroid resection. In this subgroup, one patient underwent lobectomy for monolobar goiter, one for monolobar goiter and ipsilateral intrathyroidal parathyroid, and one total thyroidectomy for multinodular goiter, with an unexpected finding of papillary thyroid carcinoma on histology. Three patients (11.1%) underwent en-bloc resection with the ipsilateral thyroid lobe for suspected PC (two for markedly elevated preoperative calcium and PTH levels and one for gland size), not confirmed at definitive hystological examination.

Among 9 patients with PC, 7 (77.88%) underwent en-bloc resection, while 2 (22.22%) had MIVAP, as malignancy was not suspected preoperatively; both declined reoperation for surgical radicalization after hystological examination.

Among 316 patients with PA, 282 (89.24%) were treated with MIVAP, while 34 (10.76%) required open parathyroidectomy. In the latter group, 19 patients had negative or discordant preoperative imaging, 3 had concomitant thyroid disease managed concurrently (two goiters and one papillary carcinoma), 2 had prior thyroidectomy, and 1 had a large parathyroid gland (diameter 4 cm) on ultrasound. In nine cases, an inadequate intraoperative PTH drop led to conversion from MIVAP to open exploration.

Operative data are reported in Table 4.

**Table 4:** Surgical approach performed in patients according to histological type

	APT *N* = 27	PC *N* = 9	PA *N* = 316
MIVAP	18 (66.66%)	2 (22.22%)	282 (89.24%)
Traditional parathyroidectomy	3 (11.11%)	0	34 (10.76%)
Traditional parathyroidectomy + thyroid resection	3 (11.11%)	0	0
En bloc resection	3 (11.1%)	7 (77.88%)	0

### Post-operative data

The average gland weight was 1700 ± 2126 (60–9000) mg, while the average diameter was 2.45 ± 1 (0.7–5) cm in APT group.

Gland weight was greater in patients with PC with a statistical difference between groups [Kruskal–Wallis test *p* < 0.001], while gland diameter was larger in those with APT with no significant statistical difference [Kruskal–Wallis test *p* = 0.008]. In terms of gland diameter, APT was comparable to PC, while regarding gland weight, APT resembled PA.

In the APT group, the average calcium level on the first post-operative day was 9.1 ± 1.075 (8.3–10.5) mg/dl, and on the second post-operative day, it was 8.55 ± 0.775 (7.5–9.90) mg/dl. The first-day postoperative calcium levels were higher in patients with PC (10.5 ± 0.975 (9.6–12), so APT exhibited a pattern similar to PA with lower first-day postoperative calcium levels [Kruskal–Wallis test *p* = 0.021]. Calcium levels on the second post-operative day are lower than calcium level on the first post-operative day in all three groups analyzed; however, they remained higher in patients with PC compared to the other two groups [Kruskal–Wallis test *p* = 0.607].

**Fig. 4:**
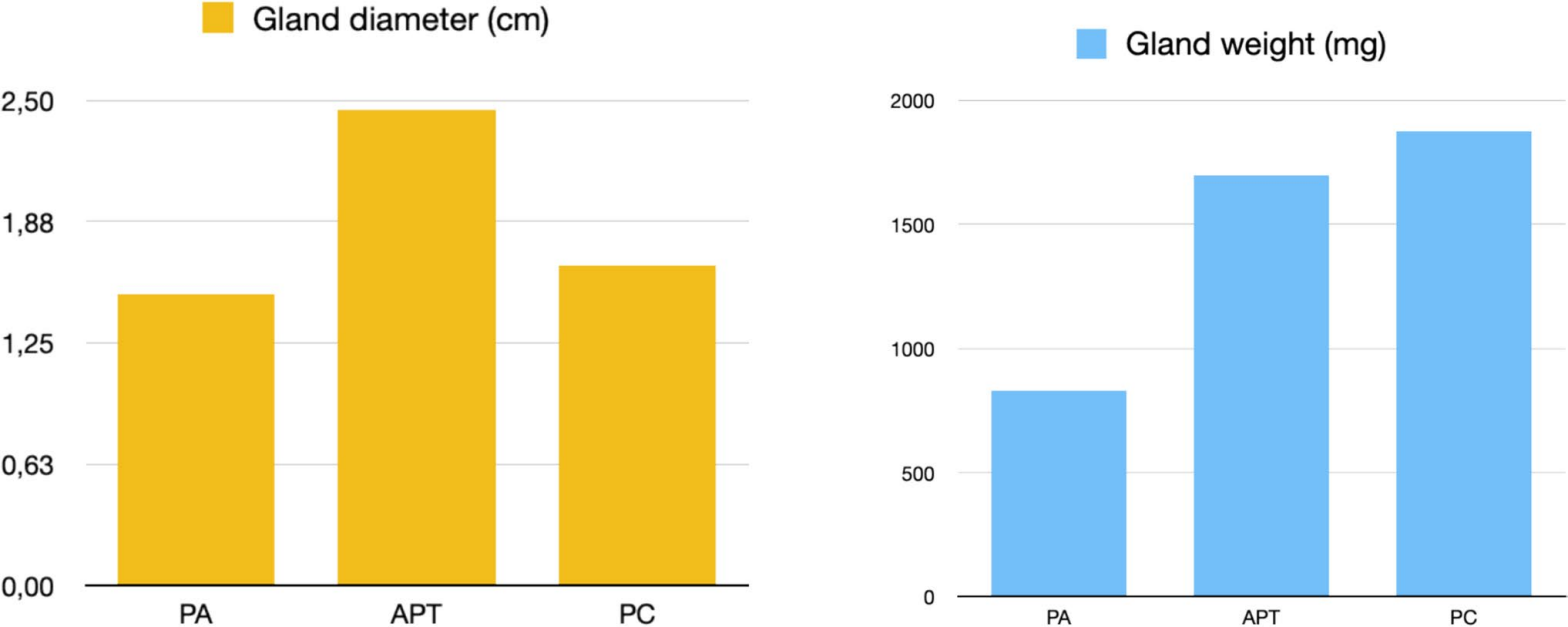
Gland weight and diameter after parathyroidectomy in patients with histologically confirmed atypical parathyroid tumor

Post-operative data are analytically reported in Table 5 and graphically represented in Fig. 4.

**Table 5:** Post-operative characteristics in the study groups

	APT *N* = 27	PC *N* = 9	PA *N* = 316
Gland weight (mg)	1700 ± 2126 (60–9000)	1875 ± 257.5 (1630–8100)	830 ± 1062.38 (60–32000)
Gland diameter (cm)	2.45 ± 1 (0.7–5)	1.65 ± 0.775 (1.3–2.6)	1.5 ± 0.2 (0.01–5.5)
I day post-operative calcium level (mg/dl)	9.1 ± 1.075 (8.3–10.5)	10.5 ± 0.975 (9.6–12)	9.1 ± 0.9675 (7.3–11.3)
*II day* post-operative calcium level (mg/dl)	8.55 ± 0.775 (7.5–9.90)	8.85 ± 0.55 (7.6–9.7)	8.8 ± 0.625 (7.41–10.5)
*Postoperative PTH level (pg/ml)*	9.1 ± 1.075 (8.3–10.5)	8.85 ± 11.075 (3.4–21)	18.6 ± 20.09 (3.4–167.8)

No statistical comparison was performed for the prevalence of Ki-67 positivity in surgical pathology specimens, as Ki-67 assessment is not routinely reported in parathyroid adenomas. However, a clear difference emerged between atypical parathyroid tumors and carcinomas. In APTs, more than 80% of cases showed a Ki-67 index < 1%, whereas in PCs all cases had a Ki-67 index ≥ 1%. Notably, 4 out of 9 carcinomas exhibited high proliferative activity, with Ki-67 values reaching 20% and 30% in 2 patients. Ki-67 data are analytically reported in Tables 6 and 7.

**Table 6:** Ki-67 proliferation index in atypical parathyroid tumors

	APT *N* = 27
Ki-67 < 1%	22
1% < Ki-67 < 2%	1
2% < Ki-67 < 3%	2
3% < Ki-67 < 4%	1
Ki-67 < 4%	1

**Table 7:** Ki-67 proliferation index in parathyroid carcinomas

	PC *N* = 9
1% < Ki-67 < 5%	5
5% < Ki-67 < 10%	2
Ki-67 = 20%	1
Ki-67 = 30%	1

### Follow up

About complications, 11 patients with APT experienced complications after surgery: 7 patients had transient hypoparathyroidism (1.9%), 1 patient had postoperative hemorrhage (0.3%), 1 patient experienced a transient recurrent laryngeal nerve injury (0.3%), and finally, 2 patients developed Hungry Bone Syndrome (0.6%). In no case of APT were there recurrence or persistence of disease. Follow-up data are analytically reported in Table 8. Chi-square test reported no inter-group differences in terms of overall or specific complications.

**Table 8:** Post-operative complications and follow-up outcomes

	APT *N* = 27	PC *N* = 9	PA *N* = 316
Transient hypoparathyroidism	7 (1.99%)	5 (1.42%)	26 (7.39%)
Permanent hypoparathyroidism	0	0	1 (0.03%)
Postoperative hemorrhage	1 (0.03%)	0	8 (2.27%)
Transient recurrent laryngeal nerve injury	1 (0.03%)	0	9 (2.56%)
Permanent recurrent laryngeal nerve injury	0	1 (0.03%)	6 (1.70%)
Hungry Bone Syndrome	2 (0.06%)	1 (0.03%)	21 (5.96%)
Recurrence	0	0	3 (0.95%)
Persistence	0	0	3 (0.95%)

No cases of disease persistence or recurrence were observed in patients with APT or PC at a mean follow-up time of 42.8 months for patients with APT and of 74.4 months for patients with PC. Only one patient affected from PC died two years after surgery, not due to recurrence, but to metastatic carcinoma of the biliary tract. Values of PTH, Calcium, Phosphorus are in the normal range for all patients, both in the group of AT and in the one of PC.

## Discussion

In our cohort of 437 patients with PHPT, we identified 27 cases of APT (6.2%) and 9 cases of PC (2.1%). These rates are higher than those generally reported in the literature for both entities. According to published data, the incidence of atypical parathyroid tumor ranges from 0.5 to 4.4% [4], while that of parathyroid carcinoma is estimated at approximately 0.5–5% [7]. This discrepancy is likely attributable to the fact that our institution is a tertiary referral center for parathyroid disorders covering a large area, managing a higher case load than other centers and consequently observing a greater incidence of these conditions.

The mean age of patients affected by APT was 64.5 ± 26 years (20–84 years), with a higher prevalence in females (67%). The pTRANI study reported a mean age at onset of APT of 58.2 ± 14.5 years and a higher prevalence in females, in agreement with our findings [7].

As highlighted by the findings from our cohort, the comparative analysis of clinical manifestations among APT, PC, and PA revealed significant differences, although within a clinical spectrum largely consistent with what has been reported in the literature on primary hyperparathyroidism (PHPT).

Among patients with APT, approximately one-third were asymptomatic, with almost all cases diagnosed incidentally (29.6%). This finding aligns with previous reports highlighting the high rate of incidental PHPT diagnoses in Western countries, facilitated by the widespread use of routine biochemical testing [8]. Particularly noteworthy is the case of a patient diagnosed through FDG-PET performed for another indication: although not a screening tool, parathyroid incidentalomas on functional imaging have been described in the literature as a rare but not negligible occurrence [9].

Renal involvement emerged as the most frequent clinical feature in our APT cohort (44.4%), exclusively presenting as symptomatic urinary lithiasis. This is consistent with current knowledge of PHPT, in which nephrolithiasis represents one of the most common and potentially disabling complications [10]. Similarly, skeletal involvement was observed in 14.8% of cases, predominantly as severe osteoporosis, with a single case of brown tumors, an entity that has become rare in countries where earlier diagnosis is common [11].

Neurological manifestations (11.1%) mainly consisted of cognitive decline, further confirming that neuropsychiatric alterations represent a possible, albeit less frequent, clinical expression of PHPT [12]. None of the patients with APT presented with gastrointestinal involvement or hypertension, features reported in the literature with highly variable frequencies [13].

When comparing APT with PC, we observed that both groups may present with asymptomatic disease or systemic complications. In PC, however, clinical severity appeared more pronounced: one patient presented to the emergency department in a soporous state due to severe hypercalcemia, a manifestation described as a paraneoplastic emergency in several case reports [14]. Furthermore, in PC we observed both brown tumors and neuropsychiatric symptoms such as depression and cognitive decline, highlighting the potentially devastating nature of the associated metabolic complications.

In summary, our data confirm that APT and PC share some clinical features with PA. They present with a higher frequency of PHPT classical complications (nephrolithiasis, osteoporosis, brown tumors) but with more severe neuropsychiatric symptoms than PA. In addition, we observed that renal complications were more frequent in patients with APT (44.44%) than in those with PC (33.33) or PA (30.68%). These findings markedly diverge from the existing literature, particularly from the recent Italian multicenter study published in the *European Journal of Endocrinology* [15], which reported that PC cases are more frequently associated with renal complications (such as nephrolithiasis or nephrocalcinosis), including a higher incidence of renal colic.

Incidental diagnosis remains common across all subgroups, but in atypical and malignant parathyroid tumors severe clinical manifestations appear to be more frequent than in PA. This finding supports the hypothesis that APT and PC may lie along a distinct clinical and biological continuum from sporadic PHPT, with important implications for surveillance and therapeutic management.

All symptomatic patients underwent parathyroidectomy in accordance with National (ISS/AME/SIOMMMS 2023 [1]) and International Guidelines (ASBMR [16] e NICE [17]) and SIUEC PDTA [18]. All the asymptomatic patients that we operated, had one or more criteria listen below and expressed in above mentioned guide lines: serum calcium levels above the upper limit of normal, with an average increase of 1.0 mg/dl (0.25 mmol/L), BMD by DXA (T-score ≤ 2.5 at lumbar spine, total hip, femoral neck or distal 1/3 radius), vertebral fracture by X-ray, CT, MRI, VFA, creatinine clearance < 60 cc/min or 24-h urine for calcium > 400 mg/d (> 10 mmol/d) and increased stone risk by biochemical stone risk analysis, presence of nephrolithiasis or nephrocalcinosis by X-ray, ultrasound or CT and finally age < 50 years.

Preoperative data in our cohort revealed that APTs displayed a biochemical profile that overlaps in some cases with adenomas and in other with carcinomas. For example, PTH levels were significantly higher in APT, yet not too far from PC, while serum calcium in APT occupied an intermediate range between PA and PC. These findings are consistent with multiple reports showing that atypical parathyroid tumors frequently demonstrate biochemical features similar to carcinomas, which complicates preoperative differentiation from true parathyroid carcinoma [19].

Patients with parathyroid carcinoma in our cohort had the highest calcium concentrations and the lowest phosphate values, concordant with the established literature describing PC as typically associated with more severe hypercalcemia and deranged mineral metabolism compared with benign disease [20].

Regarding localization studies, the higher (though not statistically significant) concordance rate observed in APT versus PA in our cohort mirrors prior reports that multimodal imaging (ultrasound combined with sestamibi SPECT/CT or 4D-CT) improves anatomic localization and surgical planning. Nevertheless, discordant or equivocal imaging remains common in routine practice and should prompt cautious operative planning—particularly when histologic features or biochemistry raise suspicion for atypical or malignant disease [21].

Our operative data highlight distinct surgical pattern among patients with APT, PC, and PA, reflecting both differences in preoperative suspicion and intraoperative management challenges.

In the APT cohort, the majority of patients (66.7%) were treated with minimally invasive video-assisted parathyroidectomy (MIVAP), confirming that, in the absence of overt clinical or radiological suspicion of malignancy, these tumors are often managed similarly to sporadic PHPT. Nonetheless, a significant minority (11.1%) required traditional parathyroidectomy according to the recommendations of the United Italian Society of Endocrine Surgery (SIUEC). In fact, in the case of suspected single-gland disease based on preoperative localization studies, a minimally invasive technique is recommended when imaging studies allows accurate localization of the pathological gland. In the absence of reliable localization, a traditional exploration or open exploration of all four glands is indicated. Furthermore, an additional subgroup underwent parathyroidectomy combined with thyroid resection, either for concomitant thyroid disease or for intrathyroidal parathyroid localization. Interestingly, in one of these cases, histology unexpectedly revealed papillary thyroid carcinoma, emphasizing the importance of systematic histopathological assessment and the potential coexistence of thyroid and parathyroid pathologies, as also described in previous studies [22].

Three APT patients (11.1%) underwent en-bloc resection with the ipsilateral thyroid lobe due to preoperative features suggestive of carcinoma, such as markedly elevated calcium and PTH levels or large gland size. This observation supports the notion that, in selected cases of atypical parathyroid tumors, clinical and biochemical parameters may raise the suspicion of malignancy and guide surgical planning toward a more aggressive approach [14]. In the pre-operative suspicion of PC, it is essential to adequately inform the patient about the risk–benefit ratio of removing the thyroid lobe, compared to not removing it at the first surgery.

As expected, the surgical management of PC showed a clear predominance of en-bloc resection (77.8%), in line with current guidelines recommending this procedure as the gold standard for ensuring adequate oncological control [23]. The two patients who underwent MIVAP had no preoperative suspicion of carcinoma and subsequently declined reoperation for surgical radicalization. This highlights a critical issue in PC management: the difficulty in achieving a correct preoperative diagnosis, with the risk of undertreatment if malignancy is not suspected before surgery [13].

In contrast, the vast majority of PA patients (89.2%) underwent MIVAP, confirming this technique as the preferred approach in sporadic PHPT due to its safety, reduced invasiveness, and favorable cosmetic outcomes. Conversion to open surgery was necessary in a small percentage (2.8%) due to an inadequate intraoperative PTH drop, reaffirming the crucial role of intraoperative PTH monitoring in guiding surgical decisions [24]. Open exploration was also required in cases of negative or discordant preoperative imaging, concomitant thyroid disease, or prior thyroidectomy conditions.

Overall, these findings confirm that surgical management of APT often lies at the intersection between PA and PC, with a large proportion of cases treated with minimally invasive techniques but a non-negligible subset requiring more extensive resections when malignancy is suspected. The overlap in surgical strategies reflects the biological continuum between atypical and malignant parathyroid tumors and underlines the importance of tailoring the operative approach to individual preoperative and intraoperative findings.

About postoperative data in our series, gland weight was significantly higher in PC, whereas gland diameter was greater in APT, resulting in an intermediate profile when compared to PC and PA. Interestingly, it was closer to PA with respect to gland weight. It is difficult to explain why atypical tumors are larger than carcinomas and we have not found any data about this in the literature. This finding might also be coincidental, considering the limited number of cases, although still notable given the rarity of the condition. A possible explanation could be that the more severe hypercalcemia observed in PC may lead to an earlier clinical presentation compared to APT, which, as a result, may have the opportunity to reach a larger size. Regarding the largest weight of the carcinoma, this may be explained by the fact that these are completely solid and compact lesions, with more fibrotic component, compared to adenoma and atypical tumors.

From a biochemical perspective, postoperative calcium kinetics differed across groups. Patients with PC showed the highest calcium levels on the first postoperative day, in line with the more severe preoperative hypercalcemia observed in this cohort and consistent with prior reports associating PC with a higher risk of postoperative hypercalcemia and incomplete biochemical remission [25]. In contrast, APT demonstrated a profile closer to PA, with lower calcium levels on the first postoperative day and a progressive normalization of calcium and PTH values. This pattern supports the concept that, despite histologic atypia, most APTs follow a postoperative course resembling that of benign lesions rather than carcinoma.

The markedly reduced postoperative PTH levels observed in APT and PC compared with PA may reflect more aggressive surgical resections in suspected malignant or atypical cases, as well as the intrinsic biology of these lesions.

Overall, our findings confirm that APT occupies a “borderline” category both morphologically and biochemically, behaving postoperatively in a manner that partially overlaps with PA and partially with PC. This underlines the clinical importance of integrating postoperative biochemical data with histopathological and molecular findings in order to optimize risk stratification and follow-up strategies.

About the Ki67 index, in our series, more than 80% of APTs displayed a Ki-67 index < 1%, while all PCs showed values ≥ 1%, with nearly half of the cases demonstrating markedly elevated levels (20–30%). This is consistent with previous reports indicating that a low Ki-67 index is typical of adenomas and atypical adenomas, whereas higher values are more frequently associated with malignancy [2]. These findings highlight the potential role of the Ki-67 proliferation index in distinguishing atypical parathyroid tumors from carcinomas. Although Ki-67 alone cannot be considered a definitive diagnostic marker, its integration with histopathological and clinical features may provide additional support in differentiating borderline lesions from overt carcinomas [26].

In our cohort, the overall postoperative morbidity associated with APT was relatively low and largely comparable to that observed in patients with PA. The most common complication was transient hypoparathyroidism (25.9%), followed by hungry bone syndrome (7.4%), postoperative hemorrhage (3.7%), and transient recurrent laryngeal nerve injury (3.7%). Importantly, no cases of permanent hypoparathyroidism or recurrent laryngeal nerve injury were recorded in the APT group.

When comparing APT with PC, postoperative complications appeared more frequently in the malignant group. More than half of PC patients experienced transient hypoparathyroidism (55.6%), and one case of permanent recurrent laryngeal nerve injury was observed. This higher complication rate is consistent with literature indicating that en bloc resections and more extensive surgery required for PC carry a greater risk of nerve injury and postoperative hypocalcemia [27]. In contrast, the complication profile of APT was more similar to PA, reinforcing its clinical behavior as an intermediate entity.

Long-term outcomes in our series were favorable. At a mean follow-up of 42.8 months for APT and 74.4 months for PC, no cases of disease persistence or recurrence were observed in either group. These results are in line with recent evidence suggesting that, although APT may harbor histological features of atypia, its clinical course is generally benign, with recurrence being rare but not negligible [4]. In PC, recurrence rates reported in the literature range from 40 to 60% over long-term follow-up [28], but our series did not record recurrence, likely reflecting the limited sample size and relatively short observation period.

Of note, biochemical normalization of calcium, phosphate, and PTH levels was consistently achieved in both APT and PC groups, confirming the efficacy of surgical management. Nevertheless, given the potential for late recurrence described in both PC and APT [29], long-term surveillance with periodic biochemical monitoring remains essential. In our study, the absence of recurrence in the carcinoma cases can be explained by the limited follow-up period, which was 74.4 months. In the literature, the average time to recurrence is around 3 years, although cases of recurrence have been reported up to 20 years after treatment [30]. The association of endocrinologists recommends follow-up with check-ups every six months for the first 5 years, and then annually for patients at low risk of recurrence, that is, those with capsular and surrounding tissue invasion. Patients at high risk of recurrence—namely those with evidence of angioinvasion, lymph node involvement (N +), and/or invasion of the trachea, esophagus, major neck vessels, and/or distant metastases (M +)—should undergo more frequent monitoring, with quarterly exams during the first 10 years, followed by exams every 6 months [31].

In summary, our follow-up data support the concept that APT behaves more similarly to PA than to PC in terms of both complication profile and long-term outcomes.

This study, however, presents several limitations. First, its retrospective design inherently restricts the strength of the conclusions that can be drawn. Second, given the rarity of the disease, the absolute number of atypical parathyroid tumors and parathyroid carcinomas remains small in the context of large populations, although the caseload appears relatively high when analyzed within a single center. Finally, all PC cases included, except one, were low risk carcinoma, according to Schulte b classification [28], which may have biased the follow-up results toward more favorable outcomes. These limitations should be taken into account when interpreting our findings.

## Conclusions

Our study confirms that atypical parathyroid tumor exhibit biochemical and pathological features overlap with both PA and PC. Nonetheless, the absence of recurrence or disease persistence in APT cases leads us to consider this tumor more similar to adenoma than to carcinoma.

The favorable evolution of atypical tumor in our case series with an average follow-up exceeding three years, could be a factor in favor of reducing the follow-up time for atypical tumors to a shorter period than the one recommended for carcinomas.

However, further multicenter and prospective studies are necessary to establish appropriate follow-up strategies for patients affected by atypical parathyroid tumors.

## Data Availability

The datasets generated and analyzed during the current study are available from the corresponding author on reasonable request.
